# Thymoquinone Inhibits the Migration and Invasive Characteristics of Cervical Cancer Cells SiHa and CaSki In Vitro by Targeting Epithelial to Mesenchymal Transition Associated Transcription Factors Twist1 and Zeb1

**DOI:** 10.3390/molecules22122105

**Published:** 2017-12-04

**Authors:** Jun Li, Md. Asaduzzaman Khan, Chunli Wei, Jingliang Cheng, Hanchun Chen, Lisha Yang, Iqra Ijaz, Junjiang Fu

**Affiliations:** 1 Key Laboratory of Epigenetics and Oncology, Research Center for Preclinical Medicine, Southwest Medical University, Luzhou 646000, China; leejun2015@foxmail.com (J.L.); asadkhan@swmu.edu.cn (M.A.K.); weichunli2013@163.com (C.W.); Jingliangc@swmu.edu.cn (J.C.); 15228233665@163.com (L.Y.); Hijaab2010@yahoo.com (I.I.); 2 State Key Laboratory of Quality Research in Chinese Medicine, Macau University of Science and Technology, Macau 999078, China; 3 Department of Biochemistry, School of Life Sciences, Central South University, Changsha 410013, China; chenhanchun@csu.edu.cn; 4 Medical College, Hunan Normal University, Changsha 410081, China

**Keywords:** thymoquinone, cervical cancer, metastasis, epithelial to mesenchymal transition, Twist1, Zeb1, E-Cadherin

## Abstract

Cervical cancer is one of the most common gynecological malignant tumors worldwide, for which chemotherapeutic strategies are limited due to their non-specific cytotoxicity and drug resistance. The natural product thymoquinone (TQ) has been reported to target a vast number of signaling pathways in carcinogenesis in different cancers, and hence is regarded as a promising anticancer molecule. Inhibition of epithelial to mesenchymal transition (EMT) regulators is an important approach in anticancer research. In this study, TQ was used to treat the cervical cancer cell lines SiHa and CaSki to investigate its effects on EMT-regulatory proteins and cancer metastasis. Our results showed that TQ has time-dependent and dose-dependent cytotoxic effects, and it also inhibits the migration and invasion processes in different cervical cancer cells. At the molecular level, TQ treatment inhibited the expression of Twist1, Zeb1 expression, and increased E-Cadherin expression. Luciferase reporter assay showed that TQ decreases the *Twist1* and *Zeb1* promoter activities respectively, indicating that *Twist1* and *Zeb1* might be the direct target of TQ. TQ also increased cellular apoptosis in some extent, but apoptotic genes/proteins we tested were not significant affected. We conclude that TQ inhibits the migration and invasion of cervical cancer cells, probably via Twist1/E-Cadherin/EMT or/and Zeb1/E-Cadherin/EMT, among other signaling pathways.

## 1. Introduction

Cervical cancer, also known as invasive cervical carcinoma, is one of the most common gynecologic malignant tumors worldwide [1,2,3], representing a serious threat to female health. Annually, about 530,000 new cases of cervical cancer are documented [4]. The American Cancer Society (ACS) estimated that 12,820 women would be diagnosed with invasive cervical cancer, and 4210 women would die from the disease in 2016 [5]. In the first edition of the National Comprehensive Cancer Network (NCCN) in 2009, the specific therapeutic methods of cervical cancer in different clinic stages are prescribed in detail, which have been widely recognized in China [6]. For cervical cancer patients, during the early stage the surgery method based on radical hysterectomy is the first-line treatment, and concomitant platinum-based chemoradiotherapy remains a curative treatment for local advanced cervical cancer, particularly for distant control of the disease [6,7]. However, the chemotherapeutic strategies are limited by their non-specific cytotoxicity and drug resistance [3,7]. Therefore, investigations to discover new and effective anti-cancer agents have gained special consideration [3].

Plant-derived natural products have been used for thousands of years in cancer treatment with very low side effects [3,8]. The seeds of *Nigella sativa* (black cumin) have a notable place in traditional medicine, mainly in Arabia, South Asia, South-East Asia, the Mediterranean, China and some African countries [8]. Black cumin seeds and oils are used for different medicinal purposes due to their activities against cancer, diabetes, hypertension, bacterial infection, and also they are known for their immunomodulatory, hepatoprotextive, kidney-protective, gastro-protective, spasmolytic, bronchodilative, anti-inflammatory and antioxidant activities [9,10,11,12]. Studies have revealed that the major phytochemical compound behind the medicinal properties of black cumin is thymoquinone (2-methyl-5-isopropyl-1,4-benzoquinone, TQ) [8,9]. TQ has been reported to target a vast number of signaling pathways in carcinogenesis in different cancers, and is hence regarded as a promising anticancer molecule [8,9,13]. EMT-inducing transcription factors (EMT-TFs) such as Twist1, Snail1, Slug, and Zeb1 play an essential role in cancer metastasis, being directly or indirectly involved in cancer cell metastasis through different signaling cascades [9,10,11,12,13,14,15,16,17], so regulating EMT-TFs might be an interesting potential approach in cancer therapeutics. Recent evidences support that TQ targets EMT-TFs to regulate metastasis in breast cancers [9]. However little is known about this in cervical cancer cells, so to clarify this further, in the current study, we assessed the cytotoxicity and anti-metastatic activities by TQ treatment and its possible mechanisms of action through different EMT-TFs in cervical cancer cell lines like CaSki and SiHa.

## 2. Results

### 2.1. Thymoquinone Inhibits Cervical Cancer Cell Growth, Migration, and Invasion

**Figure 1:**
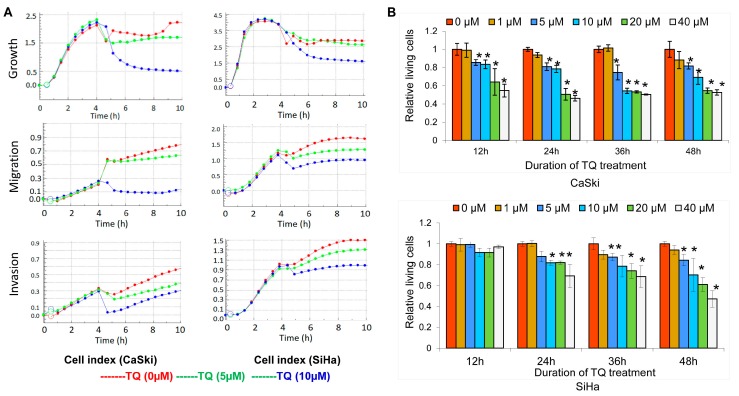
Effects of TQ on cell growth, migration and invasion in CaSki and SiHa cell lines. (**A**) Cell viability assay (CCK8 assay) also showed that treatment of TQ at a dose of 5 µM or more for 24 h or more shows significant cytotoxic effects on both CaSki and SiHa cell lines (* *p* < 0.05) (**B**).

To investigate the effects of TQ on cancer cell growth, migration and invasion, the cellular indexes were evaluated by real time cell analysis, which showed that TQ at a dose of 5 µM or more can inhibit growth, migration and invasion in both of CaSki and SiHa cells (Figure 1A).

Further we used CCK-8 analysis for a cell viability assay, which showed that TQ exerts cytotoxic activity on both CaSki and SiHa cells in a dose- and time-dependent manner (Figure 1B). After 12 h of TQ treatment, there was no clear effect of TQ on SiHa cells, but after 24 h treatment of TQ, we found significant effects of TQ, and so on after 36 and 48 h (*p* < 0.05). However, in CaSki cells, after 12 h of TQ treatment, it showed in dose dependent effects, and so on after 36 and 48 h (*p* < 0.05). These indicate that treatment of TQ at a dose of 5 µM or more for 24 h or more shows significant cytotoxic effects on CaSki or SiHa cells.

### 2.2. Thymoquinone Induces Apoptosis in Cervical Cancer Cell Lines

**Figure 2:**
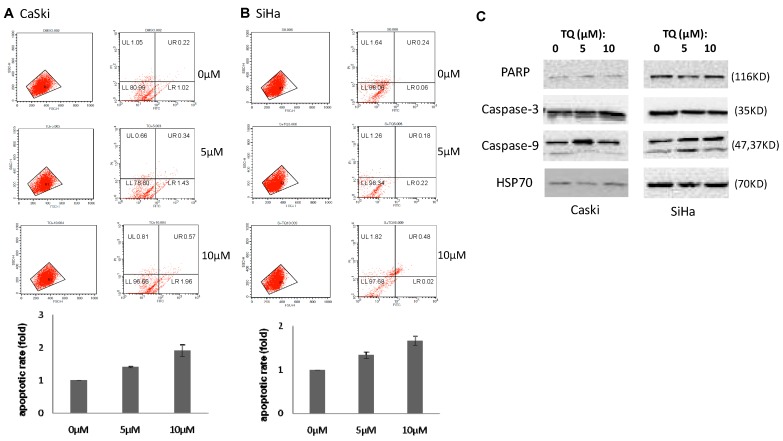
Effects of thymoquinone (TQ) on cellular apoptosis. Flow cytometric analysis shows that TQ increases the apoptotic rate in both CaSki (**A**) and SiHa (**B**) cells. Western blot for PARP, Caspase-3, Caspase-9 without or with different TQ treatment (**C**).

To evaluate whether TQ activity is related to programmed cell death, we measured the percentage of apoptotic cells in TQ-treated CaSki and SiHa cells. Annexin V and PI double staining can discriminate between apoptotic and necrotic cells. Here, flow cytometric analysis showed that TQ increases the apoptosis rate in both CaSki and SiHa cells. In contrast, the necrotic cells were reduced after treatment with TQ. The result shows that an increase in exposure dose leads to the enhancement of apoptotic cell levels (Figure 2).

### 2.3. Thymoquinone Regulates EMT Associated Genes/Proteins in Cervical Cancer Cells CaSki and SiHa

**Figure 3:**
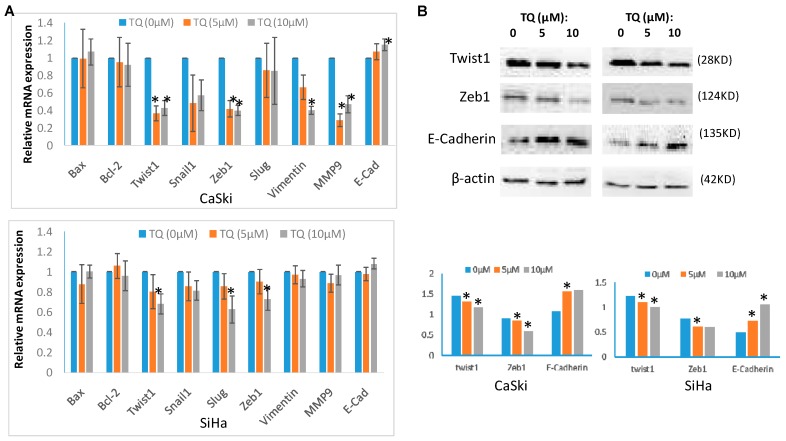
Effects of thymoquinone (TQ) on genetic expression of apoptosis and EMT associated proteins in cervical cancer cells. Real time Q-PCR analysis showed that TQ treatment (5 μM and 10 μM) for 24 h down-regulated mRNA level expression of *Twist1*, *Zeb1* both in CaSki and SiHa cells. TQ treatment up-regulated *E-Cadherin* expression too in both CaSki and SiHa cells (* *p* < 0.05). TQ also inhibits *Snail1*, *Slug*, *Vimentin* and *MMP9* in CaSki, but not in SiHa. *N-cadherin*, *Bax* and *Bcl-2* expression remained unchanged (**A**). Western blot analysis shows that TQ treatment (5 μM and 10 μM) for treatment inhibits the protein level expression of Twist1, Zeb1 both in CaSki and SiHa cells (* *p* < 0.05) (**B**).TQ treatment also significantly increased the expression of E-Cadherin in CaSki cells (* *p* < 0.05) (**B**).

The mRNA and protein levels of expression of EMT associated genes/proteins, namely Twist1, Snail1, Slug, Zeb1, E-Cadherin, N-cadherin, MMP-9 and Vimentin, as well as anti-apoptotic and pro-apoptotic proteins Bcl-2, Bax, PARP, Caspase-3 and Caspase-9, were investigated in TQ treated and non-treated cells. Both of CaSki and SiHa cells were treated with 5 μM and 10 μM of TQ for 24 h, and then total RNA was extracted from cells for quantitative RT-PCR (qRT-PCR), while DMSO treated cells were used as control. The qPCR analysis showed that TQ treatment inhibits the expression of *Twist1*, *Zeb1* expression, and increased *E-Cadherin* expression in both CaSki and SiHa cell lines (Figure 3A). TQ also affected *Snail1*, *Slug*, *Vimentin* and *MMP9* in CaSki, but the results were not consistent in SiHa. *N-Cadherin* expression was found unaffected. *Bax* and *Bcl-2* were remained unaffected (Figure 3A). Proteins in PARP, Caspase-3, Caspase-9 in CaSki and SiHa cells were also nearly unaffected (Figure 2C).

For the study of protein level expression for EMT-TFs, CaSki and SiHa cells were treated with 5 μM and 10 μM of TQ for 36 h, and proteins were extracted for western blot analysis, while DMSO treated cells were used as control. The western blot analysis showed that TQ treatment down-regulates Twist1, Zeb1 proteins and up-regulated E-Cadherinin in both CaSki and SiHa cell lines (Figure 3B).

### 2.4. Thymoquinone Directly Targets Twist1 and Zeb1 Gene

**Figure 4:**
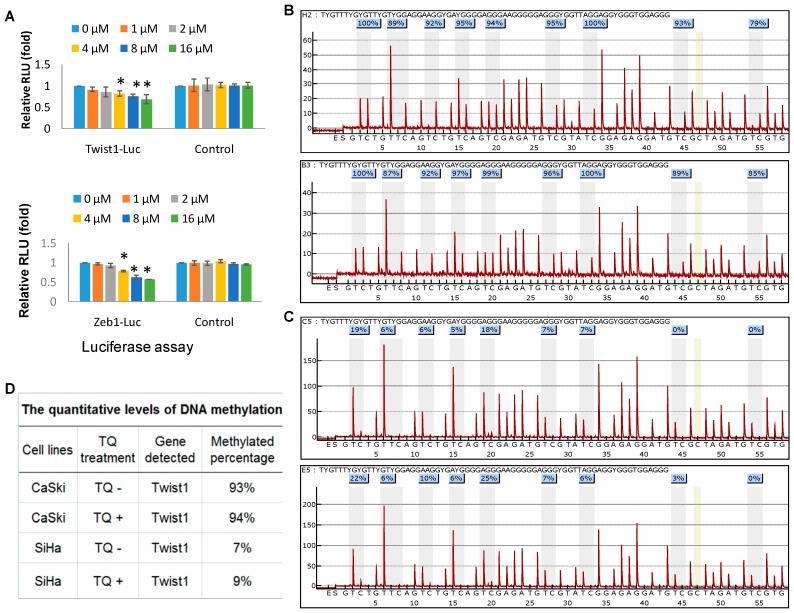
Effects of TQ on *Twist1* and *Zeb1* promoter activity. (**A**) Luciferase reporter assay shows that TQ decreased the *Twist1* and *Zeb1* reporter activity in SiHa cells, as the Relative Light Units (RLU) was decreased with the increase of TQ dosage. (* *p* < 0.05). The pyro-sequencing for the proximal promoter methylation of *Twist1* gene without and with TQ treatment in cell lines CaSki (**B**) and SiHa (**C**) were performed, and quantitative changes indicate in (**D**).

To investigate whether TQ directly targets *Twist1*/*Zeb1* genes, a luciferase reporter assay was performed. The *Twist1* and *Zeb1* reporter genes were transfected with or without TQ treatment into SiHa cell line, and after 48 h of transfection, luciferase activity was measured. Results showed that TQ dose dependently decreases the *Twist1* and *Zeb1* promoter expression respectively (relative light units or RLU, Figure 4A), indicating that *Twist1* and *Zeb1* promoter might be directly affected by TQ.

### 2.5. Effects of Thymoquinone on Twist1 Promoter Methylation in Cancer Cells

To further investigate the epigenetic mechanism whether promoter methylation affect *Twist1* expression, methylation assays for *Twist1* promoter on its CpG islands in cervical cancer cells were performed by the pyro-sequencing. The results shown in Figure 4B for CaSki and Figure 4C for the SiHa cell line indicate that the proximal promoter methylation of *Twist1* gene was found slightly increased by TQ treatment (5 μM for 24 h) (quantitative data in Figure 4D). Thus, promoter methylation of *Twist1* gene might be one of the mechanisms of *Twist1* down-regulation by TQ. However we did not test effects of TQ on *Zeb1* promoter methylation in cervical cancer cell lines due to the unavailability of this assay.

## 3. Discussion

Epithelial-mesenchymal transition (EMT) plays an important role in cancer metastasis. The transcription factors, Twist1, Snail1, Slug and Zeb1, play vital roles in initiation of EMT process [18,19,20,21,22]. Studies revealed that abnormal expression of EMT-TFs are associated with metastatic process [9,17]. Cervical cancer, which is the second most common gynecological malignant tumor in females worldwide, has a high morbidity in China, and resistance to chemotherapy is a major obstacle for effective treatment of cancers, including cervical cancer [23,24]. The acquisition of EMT features has been proposed as the key contributor of chemoresistance in cancer cells. Hence, it is crucial to obtain better insights into the mechanisms underlying the induction of EMT and to explore novel approaches to improve drug sensitivity in cervical cancer patients [25,26].

In the current study, we found that TQ has time-dependent and dose-dependent cytotoxic effects on cervical cancer cell lines. Moreover TQ dose dependently inhibited the migration and invasion processes in cervical cancer cells. The anticancer and antimetastatic activities of TQ have been previously reported in certain cancers by other studies [9,10,11,12]. However, the mechanisms of the antimetastatic role of TQ are extremely complex and still obscure, and very few studies have specifically explored the effects of TQ on cervical cancer metastasis. In this study, we found that TQ at a molecular level, decreases the expression of Twist1 and Zeb1 and increases the expression of E-Cadherin at both mRNA and protein levels. Our previous study reported that TQ inhibits metastasis via downregulation of Twist1 and upregulation of E-Cadherin in metastatic breast cancer cell lines [8,9]. Here we report for the first time the effectiveness of TQ in controlling cell growth and metastasis in cervical cancer cell lines via regulation of Twist1 and E-cadherin expression. Moreover, Zeb1 has also been found as a new target for TQ potential therapy in cervical cancer cells.

Evidences showed that Twist1 decreases the expression of E-cadhrin [19,27,28,29], and the lack of E-cadherin can induce the expression of Twist1, so forms a positive feedback, keep cells in an interstitial state, so as to induce the EMT. Like in breast cancer cells [9], in SiHa and CaSki cervical cancer cell lines, we also found by luciferase assay that *Twist1* promoter activity and expression were decreased by TQ. This indicates that *Twist1* might be a direct target of TQ. Over the past few years, Zeb1 has increasingly been considered as an important contributor to the process of malignancies including endometrial cancer, breast cancer, lung adenocarcinomas as well as cervical cancer. Li et al. [30] found that the downregulation of Zeb1 expression might reduce the proliferation and motility of cervical cancer cells. Besides, Zeb family factors (Zeb1 and Zeb2) promote EMT by repressing expression of E-cadherin [19,23,31,32]. Thus, the results of our study linked to previous studies indicating that TQ inhibits the migration and invasion of cervical cancer cells probably via Twist1/E-Cadherin/EMT or/and different Zeb1/E-Cadherin/EMT signaling pathways.

In addition, flow cytometric analysis showed that TQ increases the apoptotic rate in cells. But the expression of PARP, Caspase-3, Caspase-9, Bax and Bcl-2 in CaSki and SiHa cells were nearly unaffected by TQ, indicating that TQ effects on CaSki/SiHa apoptosis might involve other mechanisms. TQ have been previously reported by other studies, to induce apoptosis via a number of mechanisms of actions, such as modulating p53 pathway, NF-κB pathway, ROS generation etc. [9,33,34,35]. Even if literatures support the hypothesis for a role of DNA methylation in the control of *Twist1* expression, the differences treated with TQ are really too low in cervical cancer. Regulation of EMT and EMT-TFs should be involved by different pathways [36,37], as well as other epigenetic mechanisms.

It has been recently reported that many long non-coding RNAs (lncRNAs) are pivotal regulators in the oncogenesis and progression of cervical cancer [38]. For example, Ji et al. [39] reported that the HOTAIR, a lncRNA, is able to sponge miR17-5p and Battistelli et al. [40] demonstrated that this lncRNA is involved in the repression of E-cadherin expression in EMT, a typical signature observed in cancer cells, as reported by us in this study. With this regard, lncRNAs could be regulated after TQ treatment, which has been demonstrated in our previously study that co-delivery of TQ and miR-34a, a small non-coding RNA molecule, is able to enhance to inactivate EMT signaling by directly targeting *Twist1* and *Zeb1* [13]. Thus, it could be hypothesized that TQ treatment could be responsible of the reversal of EMT also through their down regulation. Future study should be performed to validate above hypothesis.

## 4. Materials and Methods

### 4.1. Cell Culture and Thymoquinone Treatment

Human cervical cancer cell lines CaSki and SiHa were cultured in RPMI1640 media (Thermo Fisher Scientific, Waltham, MA, USA) with 10% fetal bovine serum (FBS) (Pan Biotech, Bavaria, Germany). TQ was purchased from Sigma-Aldrich (St. Louis, MO, USA) and suspended in dimethyl sulfoxide (DMSO). Different concentrations of TQ were used to treat cancer cell lines, while DMSO was used as control.

### 4.2. Cell Viability Assay

Cell viability was examined by CCK-8 assay (Beyotime, Jiangsu, China). Briefly, in a 96-well cell culture plate, 5 × 10^4^ cells (in 100 μL media) were cultured per well and after incubation overnight they were treated by various concentrations of TQ (1, 5, 10, 20 and 40 μM) for 12 h, 24 h, 36 h and 48 h, respectively. At the end of incubation periods, 10 μL of CCK-8 reagent was added to each well, and kept in room temperature for 1 h. Then absorbance (optical density) was recorded at 450 nm in a microplate spectrophotometer (Multiskan™ GO, Thermo Scientific, Ratastie, Finland). The color intensity (OD values) represented the percentage of live cells in a given value.

### 4.3. Cell Growth, Migration and Invasion Assays

A real time cell analyzer (xCELLigence RTCA DP, Roche, Penzberg, Germany) was used for the real time analysis of cell migration, invasion and growth index [14,15]. 100 μL of cell suspensions (5 × 10^4^ cells/mL) were seeded on each of the 16 well E-plate for cell growth index. CMI plates were used for the analysis of cell migration and invasion, where the lower chamber wells were filled with chemotaxis inducer (10% serum supplemented media), and 100 μL of cell suspensions (5 × 10^4^ cells/mL) in serum free medium were added into the wells of upper chamber. For cell invasion assay, the membrane of the CMI plate was pre-coated with Matrigel (354277, BD Biosciences, Sparks, MD, USA) with 1:40 dilution in 1× PBS before cells were seeded. After a certain period of cell growth (usually 4 h, indicated in the figures), TQ of different concentrations (1–10 μM) were added into the wells. The process of cell migration and invasion was monitored every 30 min till the experimental endpoint.

### 4.4. RNA Extraction, RT-PCR and qPCR Analysis

After TQ treatment for 24 h, cellular total-RNA was extracted by using RNAsimple Total RNA kit (Cat No: DP419, TIANGEN, Beijing, China), following the manufacturer’s guideline. RNA concentration was measured by using ND-2000 UV/Vis spectrophotometer (NanoDrop, Wilmington, DC, USA) and final concentration was set as a 150 ng/μL for cDNA synthesis (reverse transcriptase/RT-PCR). In a 20 μL of RT reaction system, 4 μL of 5× RT buffer, 2 μL of dNTPs, 1 μL of random primer, 1 μL of Rev. Ace (enzyme, purchased from TOYOBO, New York, NY, USA and BIOBRK, Chengdu, China), 0.5 μL of super RI, 0.5 μL of RT-enhancer, 4.5 μL of RNase free water and 6.5 μL of RNA (150 ng/μL) were mixed. The reaction was completed in a thermocyler (Mastercycler Gradient, Eppendorf, Germany) with the following steps: 10 min at 30 °C, 30 min at 42 °C, 5 min at 99 °C, 5 min at 4 °C, followed by final holding at 16 °C. The synthesized cDNAs were then diluted by adding 80 μL ddH_2_O, and used as templates for quantitative PCR (qPCR) for the measurement of expression levels of *Bcl-2*, *Bax* (anti-apoptotic and pro-apoptotic proteins) and *Twist1*, *Snail1*, *Slug*, *Zeb1*, *Vimentin*, *E-Cadherin*, *N-Cadherin* and *MMP9* (major metastasis associated EMT-TF proteins) [16,17]. The sequence-specific fluorescence-labeled probes and primers for Taqman qPCR were matched by the Universal Probe Library Center (Roche) [8,14]. The primer sequences for the investigated RNA of precursor genes are presented in Table 1.

18S RNA was used as internal control. In a 10 μL of the reaction system, 5 μL of 2× PCR-master mix, 0.02 μL of probe, 1 μL of primers, 2 μL of H_2_O and 2 μL of cDNA were mixed, and reaction was completed in a StepOne plus Thermocycler (Applied Biosystem, Foster City, CA, USA) with a 40 cycle of amplification. Relative levels of mRNA expressions for each gene were obtained by normalization to 18 S RNA, and were calculated and expressed as 2^−ΔΔCT^.

### 4.5. Protein Extraction and Western Blot Analysis

After TQ treatment for 36 h, cellular proteins were extracted by using EBC lysis buffer [14]. Proteins were then separated on vertical polyacrylamide gel electrophoresis, and transferred to nitrocellulose membrane. The membrane was kept in 5% milk (in 1 × TBST) at 4 °C for 1 h, and then incubated with primary antibody solution at 4 °C for 12 h with gentle shaking. The membrane was then washed thrice with TBST, and incubated with secondary antibody tagged with horseradish peroxidase for 2–4 h at room temperature with gentle shaking. The membrane was again washed thrice with TBST, and protein bands were visualized after the chemiluminiscence reaction by using a digital imaging system (Universal Hood II, Bio-Rad Lab, Segrate, Italy) [8,14]. The primary antibodies used in this study were anti-Twist1 (Abcam), anti-Zeb1 (Cell Signaling Technology, Danvers, MA, USA), anti-PARP (#9532, Cell Signaling Technology), anti-Caspase-3 (#9665, Cell Signaling Technology), anti-Caspase-9 (#9508, Cell Signaling Technology), anti-E-cadherin (Cell Signaling Technology), anti-beta actin (Beyotime Biotechnology, Jiangsu, China), anti-HSP70 (Cell Signaling Technology). Corresponding to primary antibodies, anti-mouse (Bioworld Technology, Dublin, OH, USA) and anti-rabbit antibodies (Beyotime Biotechnology, Jiangsu, China) were used as secondary antibody. The comparative level of protein expression was measured by analyzing the visualized protein bands using the ImageJ software (National Institutes of Health, Rockville, MD, USA).

### 4.6. Luciferase Reporter Assay

Luciferase reporter assay was performed by using *Twist1* promoter reporter gene which has been reported previously [8,15]. pGL3-hZeb1-Luc promoter/reporter plasmid was constructed as follows: NM_001174093; name = ZEB1; Entrez_ID = 6935; Genome = hg38; chr10+: 31317829-31319199; TSS = 31319172; Upstream = 1343, Downstream = 27; Length = 1371; then clone it into the vector by enzymes Kpnl/Xhol. The plasmid is confirmed to be constructed as expectation by enzyme digestion and Sanger sequencing [14,41].

Then, 60%-confluent SiHa cells in 12-well plates were used to transfect with 100 ng of the pGL3-hTwist1-Luc promoter/reporter or pGL3-hZeb1-Luc promoter/reporter plasmid without or with indicated different concentration of TQ (0, 1, 2, 4, 8, 16 μM). TQ was also treated in control cells (transfected with pGL3-Basic-Luc promoter/reporter plasmid) and the activity of promoter expression of *Twist1* and *Zeb1* was measured respectively by using luciferase assay system (Promega, Madison, WI, USA). The relative luciferase activity, expressed as ‘Relative Light Units’ (RLU) was determined by using 3010 Luminometer (BD Monolight, Franklin Lakes, NJ, USA) after two days of transfection.

### 4.7. Measurement of Cellular Apoptosis

Detection of apoptosis by Annexin V binding Apoptosis detection was performed using the FITC Annexin V Apoptosis Detection Kit (BD Pharmingen, Sparks, MD, USA). CaSki and SiHa cells (10^6^ cells/mL) were plated and incubated overnight, prior to being treated with different concentration of TQ (0, 5, 10 μM). The cells were harvested, washed with PBS, re-suspended in 1 × Annexin V binding and stained with 5 μL annexin V and 5 μL PI for 10 min at room temperature in the dark. The distribution of cell populations in different quadrants was detected using BD FACSCF Calibur Cell Analyzer.

### 4.8. Twist1 Gene Methylation Assay

CaSki and SiHa cells were treated with TQ (5 μM) for 24 h, and DNA was extracted by using TIANamp genomic DNA kit (TianGen, Beijing, China).The PCR products from bisulfite-treated genomic DNA samples were analyzed with pyrosequencing technology, in order to quantify the site-specific methylation. The Qiagen bisulfite kit was used for the treatment of genomic DNA and the primers used for the amplification of *Twist1* gene promoter by PCR were as follows: F: 5′-GGGAGAGATGAGATATTATTTATTGTGT-3′; R: 5′-CTCCTCCCAAACCATTCAA-3′. The sequencing was performed using sequencing primer (5′-AGGAGGGGAAGGAAA-3′), which described previously [8]. Each site is analyzed as a C/T-polymorphism and the percentage of methylation is displayed in a small colored box just above each CpG site, where a 100% denotes a fully methylated C, a 0% denotes an unmethylated C, and intermediate C/T percentages denote partial methylation in the genomic DNA.

### 4.9. Statistical Analysis

Data was analyzed by one-way ANOVA and then post-hoc comparisons by using the SPSS v. 20 software (IBM, New York, NY, USA), and MS-Excel 2010 (Microsoft, Washington, DC, USA). Results are usually presented as mean ± SD. *p* < 0.05 was considered as significant differences.

## 5. Conclusions

Our findings suggest that TQ markedly inhibited the proliferation of cervical cancer cells in a time-dependent and dose-dependent manner and suppressed the migration and invasion of cancer cells. Targeting EMT-TFs like Twist1 and Zeb1 might be the possible mechanism of action of TQ in controlling metastasis in cervical cancer. This study indicates that TQ is as a possible chemotherapeutic agent against cervical cancer, however for the further development and establishment of TQ as a clinical drug, clinical investigations are necessary.

## Figures and Tables

**Table 1:** Primer sequences for qPCR used for mRNA isolated from human cervical cancer cells.

Genes	5′-3′ Sequences
*Q18S-48L:*	GCAATTATTCCCCATGAACG
*Q18S-48R:*	GGGACTTAATCAACGCAAGC
*Twist1-6L:*	GGCATCACTATGGACTTTCTCTATT
*Twist1-6R:*	GGCCAGTTTGATCCCAGTATT
*Snail1-11L:*	GCTGCAGGACTCTAATCCAGA
*Snail1-11R:*	ATCTCCGGAGGTGGGATG
*Slug-26L:*	TGCACCCTCGGATACCTG
*Slug-26R:*	ACATTTGGATCACAGAGGCATA
*Zeb1-31L:*	TGACTATCAAAAGGAAGTCAATGG
*Zeb1-31R:*	GTGCAGGAGGGACCTCTTTA
*E-Cadherin-84L:*	TGGAGGAATTCTTGCTTTGC
*E-Cadherin-84R:*	CGCTCTCCTCCGAAGAAAC
*Vimentin-56L:*	TGGTCTAACGGTTTCCCCTA
*Vimentin-56R:*	GACCTCGGAGCGAGAGTG
*MMP9-6L:*	GAACCAATCTCACCGACAGG
*MMP9-6R:*	GCCACCCGAGTGTAACCATA
*BCL2-2L:*	GTGGTTGGCTTACACATGGA
*BCL2-2R:*	CACCAGGGCCAAACTGAG
*BAX-55L:*	CAAGACCAGGGTGGTTGG
*BAX-55R:*	CACTCCCGCCACAAAGAT

## Abbreviations

The following abbreviations are used in this manuscript:

ACS	American Cancer Society
DMSO	dimethyl sulfoxide
FBS	fetal bovine serum
EMT	epithelial to mesenchymal transition
NCCN	National Comprehensive Cancer Network
RLU	Relative Light Units
TFs	Transcription factors
TQ	Thymoquinone
